# High spatial coherence and short pulse duration revealed by the Hanbury Brown and Twiss interferometry at the European XFEL

**DOI:** 10.1063/4.0000127

**Published:** 2021-08-23

**Authors:** Ruslan Khubbutdinov, Natalia Gerasimova, Giuseppe Mercurio, Dameli Assalauova, Jerome Carnis, Luca Gelisio, Loïc Le Guyader, Alexandr Ignatenko, Young Yong Kim, Benjamin E. Van Kuiken, Ruslan P. Kurta, Dmitry Lapkin, Martin Teichmann, Alexander Yaroslavtsev, Oleg Gorobtsov, Alexey P. Menushenkov, Matthias Scholz, Andreas Scherz, Ivan A. Vartanyants

**Affiliations:** 1 Deutsches Elektronen-Synchrotron DESY, Notkestraße 85, D-22607 Hamburg, Germany; 2 National Research Nuclear University MEPhI (Moscow Engineering Physics Institute), Kashirskoe shosse 31, 115409 Moscow, Russia; 3 European XFEL, Holzkoppel 4, 22869 Schenefeld, Germany; 4 Center for Free-Electron Laser Science, DESY, Luruper Chaussee 149, D-22761 Hamburg, Germany; 5 Department of Materials Science and Engineering, Cornell University, Ithaca, New York 14850, USA

## Abstract

Second-order intensity interferometry was employed to study the spatial and temporal properties of the European X-ray Free-Electron Laser (EuXFEL). Measurements were performed at the soft x-ray Self-Amplified Spontaneous Emission (SASE3) undulator beamline at a photon energy of 1.2 keV in the Self-Amplified Spontaneous Emission (SASE) mode. Two high-power regimes of the SASE3 undulator settings, i.e., linear and quadratic undulator tapering at saturation, were studied in detail and compared with the linear gain regime. The statistical analysis showed an exceptionally high degree of spatial coherence up to 90% for the linear undulator tapering. Analysis of the measured data in spectral and spatial domains provided an average pulse duration of about 10 fs in our measurements. The obtained results will be valuable for the experiments requiring and exploiting short pulse duration and utilizing high coherence properties of the EuXFEL.

## INTRODUCTION

With the development of pulsed lasers in the visible spectrum and the invention of X-ray Free-Electron Laser (XFEL) sources in the x-ray energy range,^1^ it has become clear that for a vast majority of experiments, essential information about the sample can only be deduced from multiple measurements. This may be accomplished by many realizations of the radiation field, i.e., multiple pulses, from these sources. For example, in single particle imaging experiments performed at XFEL sources,^2^ hundreds of thousands of coherent x-ray pulses interact with differently oriented replicas of the target particle in order to determine its three-dimensional structure with high resolution.^3–5^ It is also clear that these pulsed sources, in principle, cannot be treated as stationary ones and, hence, such properties as spatial and temporal coherence have to be revised.^6^

Coherence, at its basics, is the manifestation of correlations of the optical wave fields.^7,8^ The first order coherence or correlations of the field amplitudes may be experimentally probed by Young's double pinhole or Michelson split-and-delay type of experiments.^7,8^ Another important approach to tackle coherence is to perform the second-order intensity correlation measurements, originally proposed by Hanbury Brown and Twiss (HBT) in their pioneering experiments.^9,10^ Importantly, these experiments led to the creation of the field of quantum optics^11,12^ and became pivotal for the realization of quantum imaging and quantum technology.^13–16^

The basic idea of HBT interferometry is to explore correlation of intensities at different spatial or temporal positions, i.e., to perform measurements of the second-order correlation functions. For example, if measurements are carried out in spatial domain, this leads to the following normalized second-order correlation function:

$$g^{\left(2\right)}\left(x_{1},x_{2}\right)=\frac{\left\langle I\left(x_{1}\right)I\left(x_{2}\right)\right\rangle }{\left\langle I\left(x_{1}\right)\rangle \langle I\left(x_{2}\right)\right\rangle },$$ (1)

where $I\left(x_{1}\right)\;\;\mathrm{and}\;I(x_{2})$ are the intensities of the wave field measured at positions *x*_1_ and *x*_2_, respectively, and averaging, denoted by brackets ⟨⋯⟩, is performed over a large ensemble of different realizations of the wave field. If radiation is cross-spectrally pure and obeys Gaussian statistics,^8^ which is typical for chaotic fields, then the *g*^(2)^-function can be expressed as^17–19^

$$g^{\left(2\right)}{(x}_{1},x_{2})=1+\zeta \left(D_{\omega }\right)\cdot {\left|g^{\left(1\right)}\left(x_{1},x_{2}\right)\right|}^{2},$$ (2)

where $g^{\left(1\right)}\left(x_{1},x_{2}\right)=\langle E^{*}(x_{1})E(x_{2})\rangle /\sqrt{I\left(x_{1}\right)I\left(x_{2}\right)}$ is the first-order correlation function and $\zeta \left(D_{\omega }\right)$ is the contrast function, which depends on the radiation bandwidth $D_{\omega }$. In the limit when an average pulse duration *T* is much larger than the coherence time $\tau_{c}\;({T\gg \tau }_{c})$, the contrast function is given by $\zeta \left(D_{\omega }\right)∼\tau_{c}/T$ or inverse number of modes *M_t_* in temporal domain. In the opposite limit ${T\ll \tau }_{c}$, the contrast function reaches a constant value.^17–19^

It is especially important to understand the coherence properties of recently constructed hard x-ray FEL facilities^20–23^ as many experiments rely on the high degree of coherence of these sources. The first-order coherence properties of these sources were determined in double pinhole Young's experiments^24,25^ or by the speckle contrast analysis.^26–28^ By performing HBT experiments at XFEL sources, rich information on their statistical properties, such as the degree of spatial coherence and average pulse duration, can be determined^18,29–31^ (see for review^32^). These experiments may also shed light on the fundamental statistical properties of XFEL sources and clearly indicate whether an FEL behaves as a true single-mode laser source or rather as a chaotic source of radiation.^33^ Here, we present a statistical analysis of experimental results for the high-power European XFEL (EuXFEL) radiation by means of HBT interferometry. We performed experiments in both the spectral and spatial domains and characterized three XFEL operation modes: linear (LT) and quadratic (QT) undulator tapering at saturation, and the operation in the linear gain regime (LR). Finally, we determined the degree of coherence and characteristic pulse durations for all three regimes.

## RESULTS

**FIG. 1.**
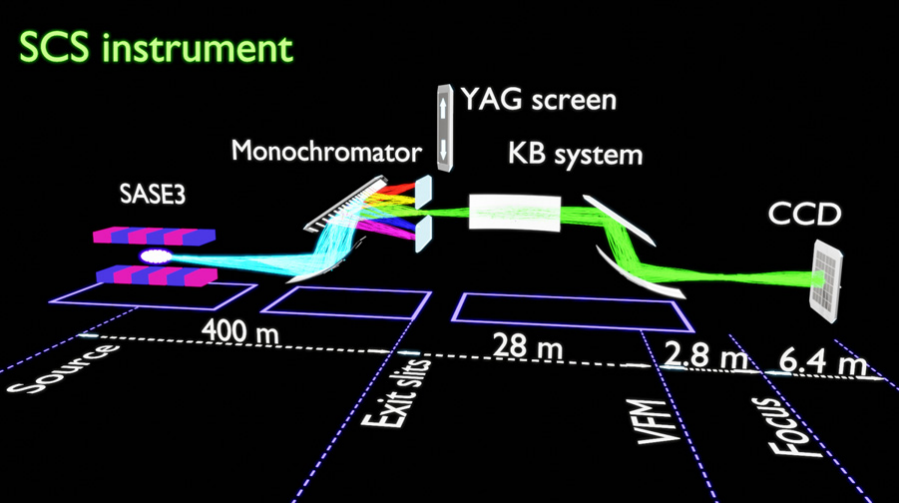
Schematic layout of the experiment. The monochromator focuses the SASE3 undulator source located about 300 m upstream onto the exit slits (ES) located 100 m downstream. Vertical focusing mirror (VFM), part of a variable bending mirrors Kirkpatrick–Baez (KB) system, located 28 m downstream from the ES refocuses the x-ray beam at 2.8 m downstream from this mirror. The measurements were taken at a distance of about 6.4 m from the focal position. Horizontal KB focusing mirror located 1.35 m upstream of the VFM refocuses the intermediate horizontal focus located at 374 m after the undulator source.

The experiment was performed at the Spectroscopy and Coherent Scattering (SCS) instrument of the European XFEL^34^ (see Fig. 1). The EuXFEL linac was operated at 14 GeV electron energy with an electron bunch charge of 250 pC in a single bunch mode at the 10 Hz repetition rate. The SCS instrument is located at the Self-Amplified Spontaneous Emission (SASE3) undulator beamline that produces intense x-ray pulses in the soft x-ray photon energy range (250 eV–3000 eV). A schematic representation of the beamline is shown in Fig. 1. The experiment was performed at 1.2 keV photon energy with three undulator configurations, i.e., LT and QT at saturation, which are dedicated settings to provide high power radiation, and a configuration for operating the EuXFEL in the LR with the average pulse energies of 1.2, 6.5, and 0.117 mJ, respectively (see the supplementary material^41^ and Fig. S1 for details).

The SCS instrument is equipped with a variable line spacing (VLS) diffraction grating monochromator.^35,36^ The monochromator was operating in the second diffraction order, corresponding to a photon energy dispersion of 2.69 eV/mm (at 1.2 keV) in the exit slit (ES) plane along the vertical direction. The experimental resolution at this energy was estimated to be better than 0.3 eV at the full-width at half maximum (FWHM) [theoretical resolution at this energy is 0.2 eV (Ref. 36)]. In the case of spectral measurements, the spectral distribution of XFEL pulses was acquired in the ES plane of the monochromator. This was achieved by introducing a YAG:Ce crystal just behind the fully opened ES and detecting the optical luminescence from this crystal by a charge coupled device (CCD) gated by a microchannel plate (MCP) detector (see the supplementary material^41^ for details).

Spatial second-order correlation measurements were performed by a back-illuminated CCD detector (Andor iKon-M 934, 1024 × 1024 pixels, pixel size of 13 × 13 *μ*m^2^) with eight pixels binned in the vertical direction and no binning in the horizontal direction. The detector was located at the end of the beamline at a distance of about 6.4 m from the x-ray beam focus. The bending of the horizontal Kirkpatrick–Baez (KB) mirror was adjusted for QT and LT/LR measurements, thus changing the horizontal size of the beam on the detector (Fig. 1). In the case of spatial correlation measurements, the bandwidth of x-ray radiation at the CCD detector was controlled by the size of the monochromator ES opening.

### Spectral analysis

**FIG. 2.**
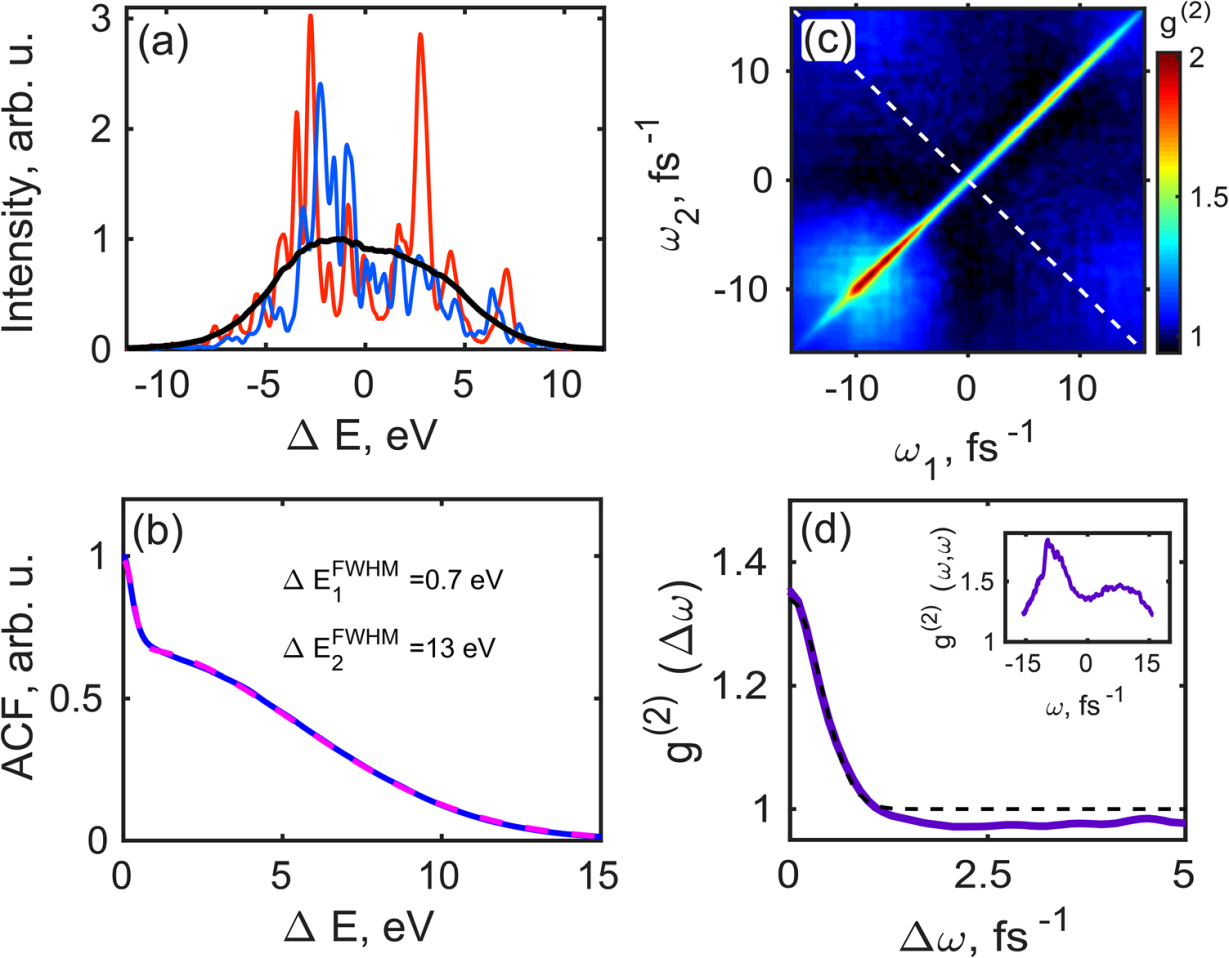
(a) Typical single shot spectra (red and blue lines) for the LT operation regime and an average spectrum for about 3 × 10^3^ pulses (black line). (b) Autocorrelation function of individual spectral lines averaged over the same number of pulses (blue solid line) and its fit with a sum of two Gaussian functions (magenta dashed line). The values of the FWHMs of these two Gaussian fits are shown in panel (b). (c) Second-order intensity correlation function of spectra $g^{\left(2\right)}\left(\omega_{1},\omega_{2}\right)$. (d) Cut along the diagonal line shown by the white dashed line in (c) and its fit (black dashed line) by the supplementary material^41^ Eq. (S16). In the inset, the profile along the diagonal $\omega_{1}=\omega_{2}$ of the $g^{\left(2\right)}\left(\omega_{1},\omega_{2}\right)$-function, shown in (c), is plotted.

Single-pulse spectra were recorded for all three regimes of the EuXFEL operation prior to spatial measurements. Each run consisted of about 3 × 10^3^ pulses for each operating condition of the EuXFEL. Below, we present the results of spectral and spatial measurements for the LT regime. As shown in Fig. 2(a) [see the supplementary material^41^ Figs. S2(a), S2(c), and S2(e) for the other operation regimes], one can observe a multimodal structure in the single pulse spectrum distribution for all operating regimes of the EuXFEL. The number of spectral modes varies depending on the operation conditions. As it is well seen in Fig. 2(a), the average spectrum does not resemble a single Gaussian function but is rather a sum of two distributions, which applies also to the other studied operating conditions. We estimated the FWHM of the average normalized spectrum directly from the experimental data and determined that it was in the range of 0.7%–1% from the resonant energy, depending on the experimental conditions [see Fig. 2(a) and Table I].

**TABLE I.** Results of the analysis in spectral and spatial domains.

Operation regime	LT	QT	LR
Photon energy, eV	1205	1205	1202
Spectral analysis			
FEL bandwidth (FWHM), eV	10.0 ± 0.1	12.6 ± 0.1	8.8 ± 0.1
Width of spectrum from autocorrelation (FWHM), eV	9.2 ± 0.1	12.0 ± 0.1	8.5 ± 0.1
Width of spike spectral lines from autocorrelation (FWHM), eV	0.49 ± 0.02	0.49 ± 0.02	0.57 ± 0.02
Coherence time (rms), as	309 ± 7	235 ± 5	316 ± 7
Pulse duration T from HBT spectral measurements (FWHM), fs	6.6 ± 2.0	5.9 ± 2.6	4.9 ± 1.2
Spatial analysis			
Average beam size (FWHM), mm	2.3 ± 0.03	1.7 ± 0.02	2.2 ± 0.03
Coherence length (rms), mm	2.4 ± 0.1	1.3 ± 0.4	2.6 ± 0.2
Degree of spatial coherence, %	91.6 ± 3	71 ± 5	89 ± 3
Pulse duration T from HBT spatial measurements (FWHM), fs	8.5 ± 1.1	12.8 ± 1.5	7.3 ± 1.2

This spectrum is about three times wider than the theoretically predicted one for the SASE3 undulator,^37^ which may be affected by the energy chirp of the beam (see simulation section in the supplementary material^41^). The knowledge of the average spectrum allows one to estimate the coherence time of x-ray radiation as $\tau_{c}=\int_{-\infty }^{\infty }{\left|\gamma \left(\tau \right)\right|}^{2}d\tau$, where $\gamma \left(\tau \right)$ is the complex degree of coherence^7,8^ [see the supplementary material^41^ Eqs. (S32) and (S33)]. We fitted the average spectrum by the sum of two Gaussian functions and determined in this way the coherence time in each operating condition of the SASE3 undulator [see the supplementary material^41^ Eq. (S35) and Table I]. For all three operation conditions, the coherence time was in the range from 200 as to 300 as.

To determine the bandwidth of single spike spectral lines, we performed the autocorrelation analysis of the spectra. The result of this analysis for the LT is shown in Fig. 2(b) [see the supplementary material^41^ Figs. S2(b), S2(d), and S2(f) for the other operation regimes]. For all three undulator settings, we observed similar features in the autocorrelation spectrum averaged over all pulses, i.e., a sharp peak (corresponding to the FEL spike width) standing on the pedestal of a broad peak (corresponding to the averaged FEL bandwidth). The values of the FWHM of both peaks, corrected for the factor of $\sqrt{2}$, are provided in Table I.

The average pulse duration in spectral domain was determined by performing correlation analysis^19^ [see the supplementary material^41^ Eq. (S11)]. The second-order correlation function in the frequency domain in the case of the LT operation is presented in Fig. 2(c) (see also the supplementary material^41^ Fig. S3 for the other operation regimes).

**FIG. 3.**
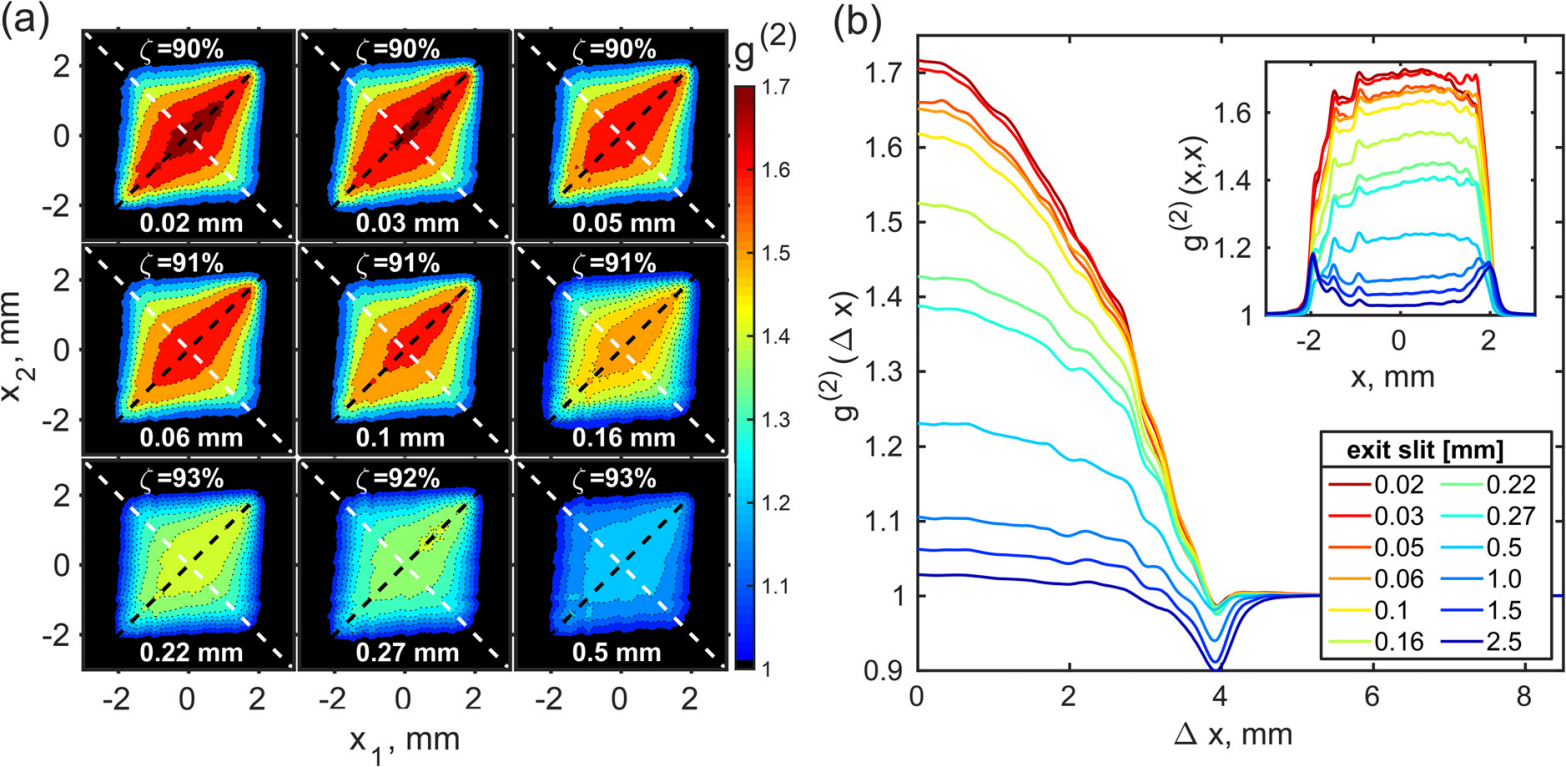
(a) Second-order intensity correlation functions $g^{\left(2\right)}\left(x_{1},x_{2}\right)$ determined in measurements in the LT mode. Each panel corresponds to a certain width of the monochromator ES, which is indicated in the panel. (b) Profiles of the $g^{\left(2\right)}(\Delta x)$-function taken along the white dashed anti-diagonal lines shown in panel (a) as a function of the ES width. In the inset, the corresponding $g^{\left(2\right)}\;(x,x)$-functions taken along the black dashed diagonal lines in panel (a) are shown.

In Fig. 2(d), we analyzed its behavior along the white dashed diagonal line shown in Fig. 2(c). Note that the contrast value is below unity for all three undulator settings due to the finite values of the degree of spatial coherence and monochromator resolution function [see the supplementary material^41^ Eqs. (S13) and (S14), as well as Ref. 19]. Fitting this profile with a function defined by the supplementary material^41^ Eq. (S16), which takes into account monochromator resolution, gave us the root mean square (rms) values *σ_T_* of an average pulse duration from which we deduced the FWHM values *T *=* *2.355*σ_T_* to be in the range from 5 to 7 fs for all three undulator settings (see Table I).

### Spatial analysis

Typical intensity distributions of individual pulses for all operation conditions, measured with small and wide ES width, are shown in the supplementary material^41^ Fig. S5. A visual inspection of the individual pulses reveals that between one and five spectral spikes were present in the XFEL beam at 2.5 mm wide ES [see the supplementary material^41^ Figs. S5(d)–S5(f)]. For further intensity correlation analysis, these intensity distributions were projected along the vertical (dispersion) direction, and correlation analysis was performed in the horizontal direction according to Eq. (1) for 1.2 × 10^4^ or 6 × 10^3^ XFEL pulses depending on the operation regime.

The average pulse intensity distribution for different monochromator ES width and typical intensity correlation *g*^(2)^*-*function for the smallest ES width of 0.02 mm for all operation conditions is shown in the supplementary material^41^ Fig. S6. We clearly observe the growth of the average pulse intensity with the increase in the monochromator ES width. We attribute the non-Gaussian shape of these intensity profiles to the slope error of the beam transport mirrors. Directly from these profiles, we estimated the FWHM of the beam size to be in the range from 1.7 to 2.3 mm, depending on the KB mirror settings and mode of the undulator operation (see Table I). As it can be seen from the supplementary material^41^ Fig. S6, the shape of the *g*^(2)^-function resembles a flat-top function. Such form of the g^(2)^-function is typical for highly coherent radiation when the coherence length of radiation is much larger than the size of the beam.^30,38^

In Fig. 3(a), the results of intensity correlation analysis for the LT mode as a function of the monochromator ES width are presented (see the supplementary material^41^ Figs. S8 and S9 for the other operation regimes). The intensity correlation functions determined along the white dashed lines in Fig. 3(a) are presented in Fig. 3(b) for different ES's width. One can see that the larger the ES width, the lower the contrast $\zeta \left(D_{\omega }\right)$ in Eq. (2) [that is the maximum value of the $g^{\left(2\right)}(\Delta x)$-function], which obeys the typical behavior predicted by this equation for a Gaussian chaotic source. We also notice that for the separation of two points of about *Δx *=* *4 mm, the correlation function $g^{\left(2\right)}(\Delta x)$ takes values below unity. We attribute these features to low values of intensity at these separations and to positional jitter of the beam.^29^

**FIG. 4.**
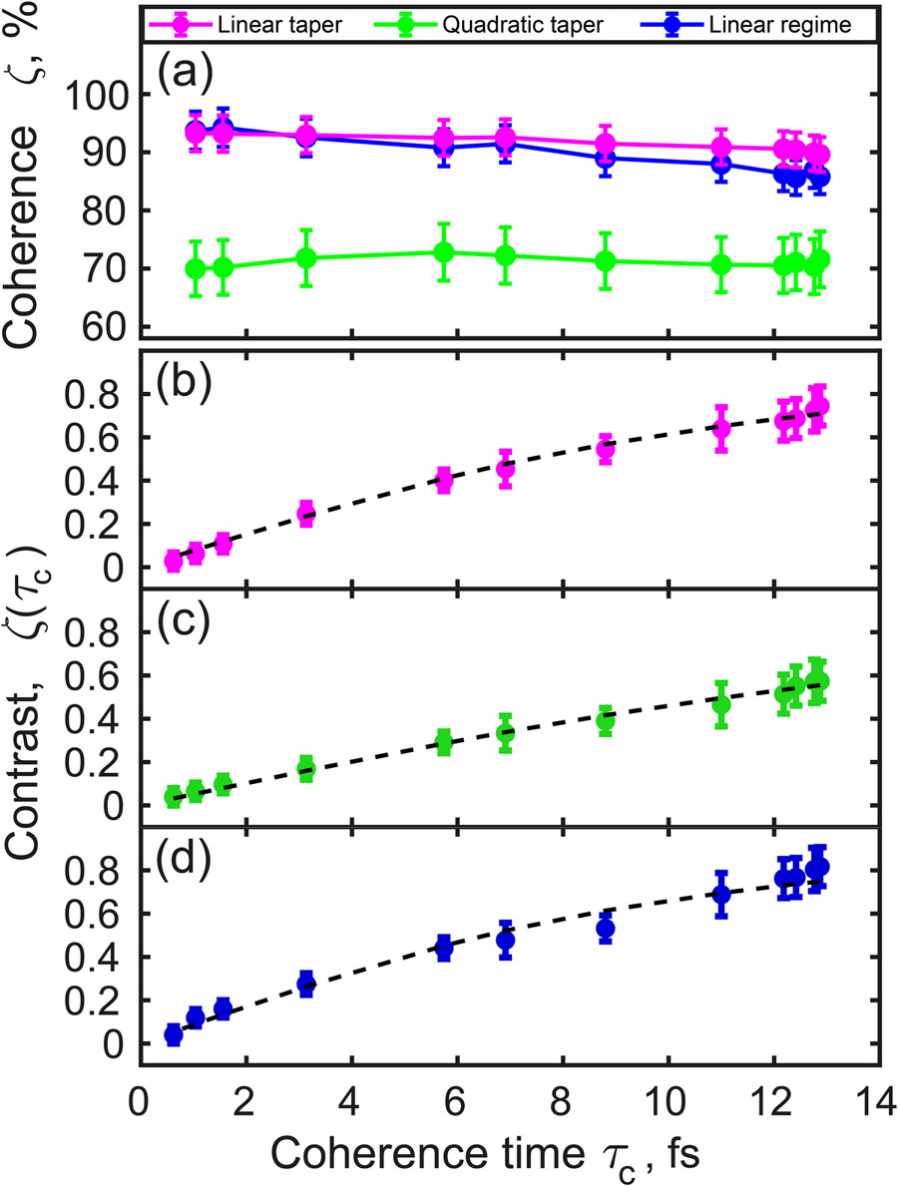
Degree of coherence ζ (a) and contrast values ζ(τ_c_) (b)–(d) as a function of coherence time for different modes of the undulator settings. (b) LT, (c) QT, and (d) LR of the undulator operation. Circles represent experimental points. Errors are calculated as the standard deviation of the data. The black dashed lines in (b)–(d) are the fit over all experimental data points according to the supplementary material^41^ Eq. (S26).

Next, we determined the degree of spatial coherence *ζ* [see the supplementary material^41^ Eq. (S29) for definition] and the coherence length *L_coh_* as a function of coherence time for all three undulator settings [see Fig. 4(a) and supplementary material^41^ Fig. S10]. The coherence time was determined according to the supplementary material^41^ Eqs. (S32) and (S33), in which the spectral density *S*(ω) was substituted by the function ${\tilde{T}}_{sl}(\omega )$ defined in the supplementary material^41^ Eq. (S21) that accounts for the finite monochromator resolution.^19^ The first observation here is that the degree of spatial coherence and coherence length essentially do not depend on the coherence time, being practically constant in the range of coherence times from 1 to 12.8 fs. Second, one can see that the value of coherence length for the LT and LR settings was about the FWHM width of the corresponding beams. For the QT, we observed slightly smaller values of the coherence length, which were also lower than the corresponding beam sizes (see Table I). From these observations, we concluded that the degree of spatial coherence reaches high values of about 85%–95% for the LT and LR modes. In the case of QT mode, we observed a slightly lower degree of spatial coherence of about 70% [see Fig. 4(a)].

We also determined the values of contrast $\zeta \left(D_{\omega }\right)=g^{\left(2\right)}\left(x,x\right)-1$ taken at *x *=* *0 as a function of coherence time for all three settings of the undulator. As it is clearly visible from Figs. 4(b)–4(d), the values of contrast strongly depend on coherence time and show the typical behavior of a chaotic source.^18,29^ One may observe in Figs. 4(b)–4(d) that the contrast values do not reach the maximum value of unity at saturation, which is due to the finite resolution of the monochromator (see Ref. 19 and the supplementary material^41^ for details). Fitting the obtained contrast values with the supplementary material^41^ Eq. (S26), provided us with the average pulse duration values from about 7 to 13 fs for the different SASE3 undulator settings of the EuXFEL (see Table I). Here, as before, we assumed the resolution value of the monochromator *R(ω*) to be 0.3 eV [see the supplementary material^41^ Eq. (S15)].

## DISCUSSION AND SUMMARY

The second-order correlation experiment performed at the high-power SASE3 undulator of the EuXFEL facility at 1.2 keV photon energy demonstrated the high degree of spatial coherence of radiation using the LT (90%) as well as operating in the LR (90%). At the same time, we observed lower values of coherence at QT (70%) undulator settings at saturation that provides highest intensity at the end station. The lower degree of spatial coherence for tapered radiation at SASE3 undulator of EuXFEL was theoretically predicted in Ref. 39. In addition to the theoretical studies, our experimental findings show that LT still provides a high degree of spatial coherence while QT degrades the spatial coherence. High values of spatial coherence for the LT and LR operation are consistent with the results of experiments performed at different XFEL sources. At the same time, we demonstrated a higher degree of spatial coherence at the EuXFEL in comparison to the other XFEL sources.^18,24–30^ This may be attributed to the higher electron energy operation of 14 GeV of EuXFEL, as suggested in Ref. 40.

By performing HBT interferometry, it was possible to determine not only the degree of spatial coherence but also the average pulse duration of radiation before the monochromator. The determined values of the pulse duration both in spectral and spatial domains were on the order from 5 to 10 fs (see Table I). These short pulse durations should be considered with the certain caution. The nominal pulse duration of the European XFEL was about 20 fs as determined from bunch length for the electron bunch measurements.^23^ The spectral bandwidth of the SASE3 undulator in our experiment was ∼1% and, thus, about two to three times larger than the baseline parameter, i.e., 0.35% at 1.2 keV.^37^ The observed broadening of the spectral profiles including individual spikes may be caused by the finite monochromator resolution as well as frequency chirp of the x-ray pulses. As soon as the broadening due to monochromator resolution is about 3%, we attribute most of the observed broadening to the frequency chirp of the x-ray pulses, which is a result of the electron bunch chirp in the accelerator modules. As it follows from our simulations (see the supplementary material^41^ for details), the spectral width, as well as the spectral spikes width, may change significantly due to the frequency chirp effects. This may cause an apparent lower pulse duration obtained from both spectral and spatial measurements from the nominal one. Thus, pulse durations upstream of the monochromator can be about two to three times longer than deduced from our analysis, lying in the range of 10–20 fs. As such, our measurements provide the lower boundary for the pulse durations of the EuXFEL at different modes of operation.

From this discussion, it is clear that additional measurements with a controlled frequency chirp of radiation will be an important step in understanding the properties of the EuXFEL radiation. It will be also important to carry out measurements using complementary methods, for example, gas ionization at the same photon energy of 1.2 keV at the Small Quantum Systems (SQS) instrument^34^ that is sharing the same undulator SASE3 at the European XFEL facility.

In summary, the statistical analysis of x-ray radiation by means of HBT interferometry is a powerful tool to understand the basic properties of the beams generated by the soft x-ray undulators at the EuXFEL. Such vital parameters as the degree of coherence and pulse duration can be determined in these experiments. The results obtained in this work will be of extreme importance for the experiments requiring and utilizing the high coherence properties and short pulse durations of the EuXFEL. We believe that methods elaborated here may become an important analysis tool for understanding statistical properties of newly developed XFELs.

## AUTHORS' CONTRIBUTIONS

I.A.V. designed the study. M.S. operated the SASE3 undulator. N.G., G.M., L.le.G., B.K., M.T., A.Y., and A.S. operated the SCS beamline. R.K., D.A., J.C., L.G., A.I., Y.Y.K., R.P.K., and D.L. performed data acquisition. R.K. performed data analysis. R.K. and I.A.V. wrote the manuscript. All authors read and approved the final version of the manuscript.

## Data Availability

The data that support the findings of this study are openly available in Zenodo.org at http://doi.org/10.5281/zenodo.5186442, reference number https://zenodo.org/deposit/5186442#.
